# Evaluation of Blood Cultures from SARS-CoV-2-Positive and Negative Adult Patients †

**DOI:** 10.3390/healthcare11182581

**Published:** 2023-09-19

**Authors:** Bahar Akgün Karapınar, İlvana Çaklovica Küçükkaya, Yasemin Bölükbaşı, Sertaç Küçükkaya, Gonca Erköse Genç, Zayre Erturan, Ali Ağaçfidan, Betigül Öngen

**Affiliations:** 1 İstanbul Faculty of Medicine, Medical Microbiology-Bacteriology Department, İstanbul University, 34093 İstanbul, Turkey; ilvanacaklovica@istanbul.edu.tr (İ.Ç.K.); yaseminbolukbasi@istanbul.edu.tr (Y.B.); sertac.kucukkaya@istanbul.edu.tr (S.K.); ongenb@istanbul.edu.tr (B.Ö.); 2 İstanbul Faculty of Medicine, Medical Microbiology-Mycology Department, İstanbul University, 34093 İstanbul, Turkey; gonca.genc@istanbul.edu.tr (G.E.G.); zerturan@istanbul.edu.tr (Z.E.); 3 İstanbul Faculty of Medicine, Medical Microbiology-Virology and İmmunology Department, İstanbul University, 34093 İstanbul, Turkey; ali.agacfidan@istanbul.edu.tr

**Keywords:** blood culture, SARS-CoV-2, COVID-19, co-infection, antimicrobial resistance

## Abstract

Bacteremia and fungemia are significant causes of morbidity and mortality that frequently occur as co-infections with viral respiratory infections, including SARS-CoV-2. The aim of this study was to evaluate the microorganisms that were isolated from the blood cultures of SARS-CoV-2-positive and negative patients and investigate their antimicrobial resistance patterns. A retrospective analysis was performed of 22,944 blood cultures sent to the laboratory between November 2020 and December 2021. Blood culture analyses were performed using the BD Bactec FX automated system. Identification was carried out using conventional methods, namely, VITEK-2 and MALDI-TOF MS. Antibacterial/antifungal susceptibility tests were performed according to EUCAST/CLSI recommendations. SARS-CoV-2 tests were performed with RT-PCR. Culture positivity was detected in 1630 samples from 652 patients. Of these 652 patients, 633 were tested for SARS-CoV-2; 118 (18.6%) were positive and 515 (81.3%) were negative. The bacteria and fungi that were isolated at the highest rate in SARS-CoV-2-positive patients were methicillin-resistant coagulase-negative staphylococci (MR-CoNS) (21.5%), *Escherichia coli* (12.4%), *Klebsiella pneumoniae* (12.4%), *Candida albicans* (1.65%), and *Candida glabrata* complex (1.65%), while in the negative patients, the highest rates were for *E. coli* (21.3%), MR-CoNS (13.5%), *K. pneumoniae* (12.05%), *C. albicans* (2.1%), *Candida parapsilosis* (1.1%), and *Candida tropicalis* (0.9%). No statistically significant difference was determined between COVID-19-positive and negative patients in terms of detection, such as with the *Pseudomonas* spp., *Enterococcus* spp., and methicillin-resistant *Staphylococcus aureus* isolated from the blood cultures (*p* > 0.05). The most common isolate was MR-CoNS in SARS-CoV-2-positive patients (*p* = 0.028). *Acinetobacter baumannii* was more frequent (*p* = 0.004) and carbapenem-resistant *K. pneumoniae* was isolated at a higher rate (60% vs. 43%) in SARS-CoV-2-positive patients compared to SARS-CoV-2-negative patients (*p* > 0.05). These findings highlight the fact that isolation procedures should not be disregarded and the distribution of bacterial/fungal agents of bloodstream infections and their antibiotic resistance should be followed up during a pandemic, such as in the case of COVID-19.

## 1. Introduction

Viral respiratory tract infections cause high rates of morbidity and mortality throughout the world. Of the six largest outbreaks in the world in the last 20 years, four were caused by respiratory tract infections. Morbidity and mortality caused by bacterial, viral, and fungal infections are the main complications of viral infections, especially with viruses affecting the respiratory tract. As SARS-CoV-2 causes severe lung infections, similar to those seen in deaths by bacterial co-infection in the H1N1 influenza pandemic, the monitoring of morbidity and mortality is important [1]. SARS-CoV-2 infection can also occur as a co-infection secondary to bacterial infection or bacterial super-infection and may develop secondary to SARS-CoV-2 infection, depending on the host, virus, and bacterial factors [2]. Data have shown that culture-proven infections occur in 4–15% of hospitalized COVID-19 patients and are significantly associated with mortality [3].

SARS-CoV-2 infection may cause tissue destruction with the development of a combined infection that increases bacterial colonization and attachment in the relevant region. Airway dysfunction, cellular pathology, and tissue destruction are induced by the presence of SARS-CoV-2 and/or bacterial co-infection and can facilitate the systemic spread of pathogens, thereby significantly increasing the risk of bloodstream infections and sepsis [2]. Bloodstream infections (BSI) in COVID-19 patients may be associated with the systemic dissemination of co-pathogens caused by SARS-CoV-2-induced tissue destruction [2]. In severe COVID-19 lower respiratory tract infections, multidrug-resistant (MDR) bacteria such as *Pseudomonas* and *Acinetobacter* can colonize this damaged area and trigger secondary infections [4]. The risk of bacterial infection may be higher in patients with severe laboratory and clinical conditions. Co-infections or secondary infection positivity can vary between 0.6% and 45%. However, further studies involving bacterial species and infection sites are needed for detailed evaluations [5].

In patients with severe febrile illness, blood cultures are still essential for clinicians to be able to rule out bacterial/fungal BSI infections. However, there is a lack of sufficient data on the prevalence of bacterial and fungal agents causing BSI in patients infected with SARS-CoV-2 [6,7]. Recent studies have shown *Escherichia coli*, coagulase-negative staphylococci (CoNS), *Klebsiella pneumoniae*, methicillin-resistant *Staphylococcus aureus* (MRSA), and methicillin-sensitive *Staphylococcus aureus* (MSSA) to be the most frequently isolated agents in BSI [6,7]. The frequent use of more than one antimicrobial drug due to viral pneumonia and secondary infection results in an increased consumption of antimicrobials [8]. In addition, factors such as patient density, a prolonged stay in the ICU, the use of mechanical ventilators, and the expanded use of antimicrobials have resulted in the emergence and rapid spread of MDR bacteria [5,9]. Pathogens displaying a multidrug-resistant phenotype, such as carbapenem-resistant *Enterobacterales* (CRE), can cause problems in antimicrobial treatment processes [3]. Therefore, the determination of susceptibility and the prevalence of bacterial co-infections are likely to provide important information regarding the need for and choice of antibiotics [6].

Studies conducted during the COVID-19 pandemic on the distribution of microorganisms causing infections, the frequency of polymicrobial infections, and the effect of the pandemic on changes in antimicrobial resistance are important for ongoing and future processes. Therefore, the aim of this study was to evaluate the bacteria and fungi isolated from blood cultures of SARS-CoV-2-positive and negative patients, to investigate antimicrobial resistance patterns, and to compare the findings of these two patient groups in respect of the rates of bacteremia and fungemia that were determined.

## 2. Materials and Methods

### 2.1. Study Population

A retrospective analysis was performed with a total of 22,944 blood cultures sent to the laboratory between November 2020 and December 2021. When defining BSI, if a species belonging to the skin flora was found among the isolated microorganisms, it was considered that the same species should be recovered in at least 2 blood cultures collected from the same patient within 24 h, according to the Centers for Disease Control and Prevention (CDC) criteria [10]. The growth of these microorganisms in a single blood culture within 24 h was considered contamination, except in the neonatal period [11].

### 2.2. Blood Culture

Blood cultures were performed using the BD Bactec FX (Becton Dickinson, Sparks, MD, USA) automated system and cultures were incubated for up to 5 days. In special circumstances (for *Brucella* spp., etc.), the cultures were incubated for longer than in the standard procedure [12]. All positive blood cultures were subcultured on 5% Columbia sheep blood agar (Becton Dickinson, USA) and incubated at 35–37 °C under a 5–10% CO_2_ atmosphere for 48 h. Anaerobic positive blood cultures were also subcultured on anaerobic media and incubated at 35–37 °C under anaerobic conditions for 48 h. If there was no growth on the initial media, the blood cultures were subcultured on chocolate agar (incubated at 35–37 °C under 5–10% CO_2_ atmosphere for 48 h) for the isolation of fastidious microorganisms and on blood agar (incubated micro-aerobically at 35–37 °C for 48 h) for *Campylobacter* species.

#### 2.2.1. Identification of Bacterial and Fungal Isolates

Bacterial identification was performed using conventional methods and with the VITEK-2 Compact system (bioMérieux, Marcy l’Etoile, France). Fungi isolated from Myco F or aerobic bottles were identified by morphological examination on cornmeal agar with Tween 80 and API ID 32C (bioMérieux, Marcy l’Etoile, France). Incompatible results were confirmed via MALDI-TOF MS (bioMérieux, Marcy l’Etoile, France).

#### 2.2.2. Antimicrobial Susceptibility Testing

The antibacterial susceptibilities of the isolates were investigated using the standard Kirby–Bauer disk diffusion method and the VITEK-2 Compact system (bioMérieux, France) when necessary. Antifungal susceptibility was tested with the gradient test method using Roswell Park Memorial Institute medium (RPMI-1640) (Sigma-Aldrich, St. Louis, MO, USA), in order to detect the minimum inhibitory concentration (MIC) values based on elliptical growth around the antifungal gradient. The values were read on the higher MIC values side of the strips. *Candida parapsilosis* ATCC 22019 and *Candida krusei* ATCC 6258 were used for susceptibility testing as quality-control isolates. Antibacterial and antifungal susceptibility tests were performed and evaluated in accordance with the EUCAST/CLSI criteria [13,14,15,16]. “Intermediate (I): susceptible, increased exposure’’ strains of bacteria were considered susceptible. Since there are no clinical breakpoints for *Candida auris*, the MICs were evaluated according to the tentative breakpoints determined by the CDC [17].

### 2.3. The COVID-19 Diagnoses of the Patients

COVID-19 status was confirmed via SARS-CoV-2 real-time polymerase chain reaction (RT-PCR) test positivity in the nasopharyngeal and oral swabs.

### 2.4. Statistical Analysis

The software SPSS for Windows version 15.0 was used for statistical analysis. Descriptive statistics were given as numbers and percentages for categorical variables. Proportions were compared with the chi-square (χ^2^) test in independent groups. The Pearson chi-square test was used when there were no cells with expected values of < 1 and the number of cells with values of <5 was a maximum of 20%. When these conditions were not met, either Fisher’s exact test results for 2 × 2 tables were given, or Monte Carlo simulation results for 2 × 3 tables. Tests were performed within a 95% confidence interval. A value of *p* < 0.05 was considered statistically significant.

### 2.5. Ethical Approval

The research was approved by the University of Health Sciences İstanbul Training and Research Hospital Ethics Committee (protocol code: 63 and date of approval: 11 February 2022).

## 3. Results

The hospital where the study was conducted is a single-center university hospital with a burns unit, hematological units, an organ transplant center, and a bed capacity of 1183, of which 118 are intensive-care beds, with the hospital serving patients from different regions of Turkey, mostly from Istanbul.

Blood culture analysis was performed in the microbiology laboratory of our hospital. From a total of 22,944 blood cultures taken during the study period, culture positivity was determined in 1630 samples from 652 patients. During this process, a total of 200,180 SARS-CoV-2 tests were performed, of which 29,318 were positive and 170,862 were negative. Culture positivity was detected in 652 patients (321 outpatients and 331 inpatients) and 633 were tested for SARS-CoV-2; 118 were positive (18.6%; 45 females, 73 males; mean age: 62.7 years) and 515 were negative (81.3%; 247 females, 268 males; mean age: 61.7 years).

Internal medicine outpatients comprised 32% of the SARS-CoV-2-positive and 47% of the SARS-CoV-2-negative patients. A statistically highly significant difference was detected between the SARS CoV-2-positive and negative patients in the ICU (*p* < 0.001). The distribution of patients in the clinics and the ICU where the blood samples were collected is given in Table 1.

A total of 671 pathogens were isolated from the blood cultures of the patients. Of these, 252 were fermentative Gram-negative rods, 66 were non-fermentative Gram-negative rods, 300 were Gram-positive cocci, 3 were Gram-positive rods, 7 were anaerobic bacteria, and 5 were other bacterial species (*Listeria monocytogenes* (n:2), *Campylobacter coli* (n:1), *Campylobacter jejuni* (n:1), and *Moraxella nonliquefaciens* (n:1)), totaling 633 bacteria (94.3%). Fungi were isolated from the blood cultures of 38 (5.7%) patients. The isolated microorganisms are shown in Table 2 and Table 3.

**Figure 1:**
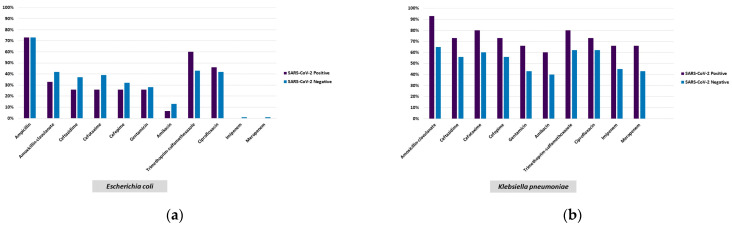

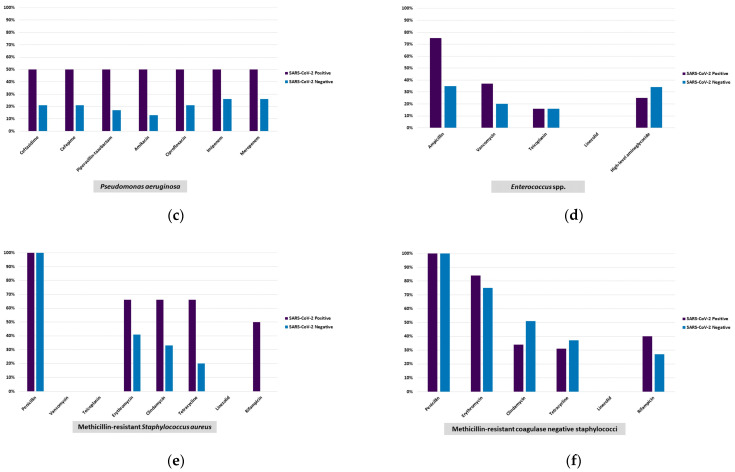
Antibiotic resistance profiles of the most frequently isolated bacteria. (**a**) *Escherichia coli*; (**b**) *Klebsiella pneumoniae*; (**c**) *Pseudomonas aeruginosa*; (**d**) *Enterococcus* spp.; (**e**) methicillin-resistant *Staphylococcus aureus*; (**f**) methicillin-resistant coagulase-negative staphylococcus.

The microorganisms isolated at the highest rate in SARS-CoV-2-positive patients were methicillin-resistant coagulase-negative staphylococci (MR-CoNS) (21.5%), *E.coli* (12.4%), *K. pneumoniae* (12.4%), *Candida albicans* (1.65%), and the *Candida glabrata* complex (1.65%), and in negative patients, they were *E. coli* (21.3%), MR-CoNS (13.5%), *K. pneumoniae* (12.05%), *C. albicans* (2.1%), *C. parapsilosis* (1.1%) and *Candida tropicalis* (0.9%). *A. baumannii* was detected at the rate of 5.8% in SARS-CoV-2-positive patients and at 1.1% in SARS-CoV-2-negative patients (*p* = 0.004). *E. coli* was more common (*p* = 0.026) in the PCR-negative group, while MR-CoNS were detected at a higher rate in the PCR-positive group (*p* = 0.028) (Table 2). No statistically significant difference was determined between the COVID-19-positive and negative patients in respect of *Pseudomonas* spp., *Enterococcus* spp., and methicillin-resistant *Staphylococcus aureus* isolated from the blood cultures (*p* > 0.05) (Table 2). Polymicrobial growth was determined in the cultures of 17 patients, of which 5 were SARS-CoV-2 positive (Table 4). The rate of carbapenem-resistant isolates among *K. pneumoniae* strains was 60% in SARS-CoV-2-positive patients, and 43.75% in negative patients (*p* > 0.05) (Table 2, Figure 1). No statistically significant difference was determined between the COVID-19-positive and negative patient groups with respect to the antibiotic susceptibility rates of the microorganisms detected (*p* > 0.05) (Figure 1). Resistance to imipenem and meropenem was determined in 13 of the 14 *A. baumannii* isolates, and of these 13 strains, 6 were from the cultures of SARS-CoV-2-positive patients, 5 of whom were being treated in the ICU (Table 4).

In the detection of MR-CoNS, MS-CoNS, *E. coli*, and *K. pneumoniae,* 45% (46/102), 34.7% (17/49), 40.4% (53/131), and 36.3% (29/80) were isolated from oncology/hematology patients, respectively. No underlying airway or other infections were detected in SARS-CoV-2-negative patients from whom *E. coli* was isolated (Table 4).

The three most frequently isolated species of fungi were *C. albicans* (39.47%; n:15), the *C. parapsilosis* complex (18.42%; n:7), and *C. tropicalis* (15.78%; n:6), and *C. auris* was isolated from one COVID-19 patient in the ICU [18]. Susceptibility tests were performed for 15 of the isolated fungi against antifungals requested by the clinician (Table 3). Of the seven *C. parapsilosis* complex isolates, three isolates were tested for antifungal susceptibilities, and in two, resistance to fluconazole was detected. Susceptibility to posaconazole and voriconazole was determined in three *C. albicans* isolates. One isolate was detected as resistant to voriconazole and three had MIC values above the ECVs for posaconazole. The *C. auris* isolate was resistant to fluconazole, and amphotericin B (Table 3).

A statistically significant difference was detected in the polymicrobial growth rates of SARS-CoV-2-positive and negative patients (*p* = 0.006) (Table 5).

## 4. Discussion

The COVID-19 pandemic imposed a devastating burden on healthcare systems around the world [19]. By affecting the epidemiology of other infections, the pandemic may have been reflected in healthcare services in the form of altered courses of bacteremia and fungemia. As a matter of fact, in recent studies examining the distribution of bacterial and fungal agents as well as their resistance profiles in BSI in COVID-19 patients; it has been stated that the agents isolated from COVID-19 patients were the organisms that most likely reflected the commensal skin microbiota at a high rate [20,21].

In the present study, the number of patients hospitalized in the ICU was 120, of whom 37 were SARS-CoV-2-positive. In another study performed during the pandemic period, although *Enterococcus* spp., *Staphylococcus aureus*, *K. pneumoniae*, and *C. albicans* were found at higher rates compared to the pre-pandemic period, community-acquired BSI cases were reported to be higher in individuals who were SARS-CoV-2-negative (15.8 per 1000 admissions) than those who tested positive (9.6 per 1000 admissions) [22]. In our study, microorganisms were detected in 79.1% and 18% of the blood cultures of SARS-CoV-2-negative and positive patients, respectively. In addition, lower rates of fermentative Gram-negative bacilli and *E. coli* (*p* = 0.013, *p* = 0.026, respectively), and higher rates of *A. baumannii*, MR-CoNS, *Rhizobium radiobacter*, and *C. glabrata* complex (*p* = 0.004, *p* = 0.028, *p* = 0.034, *p* = 0.034, respectively) were found in SARS-CoV-2-positive patients compared to SARS-CoV-2-negative patients in all clinics. In parallel to the other studies, MR-CoNS (21.5%) was isolated at the highest rate from the blood samples of SARS-CoV-2 patients. In a study by Michailides et al. [23] of patients with COVID-19 infection, CoNS, and *K. pneumoniae*, together with *A. baumannii*, were the most frequently isolated bacteria in early and late (> 5 days) nosocomial bacterial infections, respectively. Bahceci et al. [24] isolated CoNS (31%) and *A. baumannii* (27.5%) at higher rates. Michaelides et al. [23] stated that a prolonged hospital stay may increase CoNS isolation due to the development of superinfections. In the current study, the MR-CoNS (21.5%) and MS-CoNS (9.9%) isolation rates were found to be higher in patients determined to be SARS-CoV-2-positive. The blood culture contamination rate in our laboratory was 3%. The isolation rate (21.5%) of MR-CoNS, accepted as a pathogen according to the CDC recommendations [10], is very high in the current study compared to the contamination rate detected in the previous results of our laboratory. The higher isolation of CoNS in blood cultures from COVID-19-positive patients may be a result of possible concern felt by the staff during the collection of the sample, which was conducted in a stressful environment with isolation precautions on a COVID-19 ward.

Segala et al. [25] reported higher incidence rates of nosocomial BSI related to *S. aureus* and *Acinetobacter* spp. in the pre-pandemic period among COVID-19-negative patients in wards, compared to COVID-19-positive patients who were hospitalized in ICUs during the pandemic period [0.3 (95% CI 0.21–0.32) and 0.11 (0.08–0.16) new infections per 100 patient/day, respectively] but a 48% lower incident risk of *E. coli* infections in COVID-19-positive wards. In the current study, the *E. coli* isolation rate was found to be higher in patients who were SARS-CoV-2-negative (*p* = 0.026), and no underlying airway or other infection was detected in SARS-CoV-2-negative patients from whom *E. coli* was isolated (Table 4).

The presence of bacterial and fungal co-infections has been reported to increase the mortality of patients with severe COVID-19 [22,25]. CoNS (OR: 25.39), non-*albicans Candida* species (OR: 11.12), *S. aureus* (OR: 10.72), *Acinetobacter* spp. (OR: 6.88), *Pseudomonas* spp. (OR: 4.77), and *C. albicans* (OR: 3.97) have been isolated from these cases [26].

Taking into account the importance of antimicrobial management in preventing the emergence of antimicrobial resistance, an assessment of the prevalence and epidemiological characteristics of bacterial co-infection is crucial in the guidance of the appropriate empirical antibiotic therapy in the presence of an infection. Antimicrobial drugs can be prescribed either prophylactically or preemptively, especially for ICU patients. In a study of ICU patients hospitalized in ICUs from 88 different countries, despite the suspicion or the presence of bacterial co-infection in only 54% of the patients, treatment or prophylaxis with at least one antibiotic was administered in 70% of cases [27]. In another study conducted on COVID-19 patients, antibiotics were prescribed for 72% of the patients, although only 8% had confirmed bacterial or fungal co-infection [27]. The improper use of antibiotics may also lead to the emergence of resistance in bacteria and side effects in patients.

According to the COVID-19 special report published by the CDC in 2022, which investigated the impact of COVID-19 on antimicrobial resistance, there was a 35% increase in carbapenem-resistant *Acinetobacter* infections compared to 2019 and 2020 and a 78% increase in nosocomial infections, while a 35% increase in CRE infections was reported [28]. Mahmoudi et al. [29] found the resistance rates of co-trimoxazole, piperacillin, ceftazidime, and cefepime to be 74%, 67.5%, 47.5%, and 42.5% in *Enterobacteriaceae* strains and 90% for oxacillin, erythromycin and clindamycin in *S. aureus*, respectively. The sensitivity of imipenem in *P. aeruginosa* was 90% isolated from COVID-19 patients.

This situation shows that the isolation rates of carbapenem-resistant *K. pneumoniae* and *A. baumannii* strains should not be overlooked in the context of healthcare-associated infections in SARS-CoV-2-positive patients [30,31]. Following up the antibiotic resistance rates has been of great importance due to the increase in the isolation of multidrug-resistant strains. In a study of COVID-19-positive patients, 48% (*n* = 38/79) of *S. aureus* and 40% (*n* = 10/25) of *K. pneumoniae* isolates were found to be resistant to methicillin and carbapenems, respectively [25]. However, in the current study, MR-CoNS was isolated from SARS-CoV-2-positive patients at a significantly higher rate (21.5%) but no statistically significant difference was detected in the isolation rate of *S. aureus* and the methicillin resistance rate of the isolates between COVID-19-positive patients and SARS-CoV-2-negative individuals. In another study, meropenem resistance in *K. pneumoniae* strains isolated from patients in the ICU was reported to increase from 79.8% in 2019 to 92.4% in 2022. Moreover, the meropenem resistance rates of *A. baumannii* were determined to increase from 92.6% in 2018 to 97.9% in 2022 in the ICU and from 82.3% to 91.6% in the wards (*p* < 0.001) [30]. In the current study, the imipenem resistance of *K. pneumoniae* isolates was found to be 66.7% and 45.3% (*p* = 0.137), whereas meropenem resistance was 66.7% and 43.8% (*p* = 0.110) in SARS-CoV-2-positive and negative individuals, respectively. A great majority of the *A. baumannii* isolates were resistant to imipenem and meropenem in both SARS-CoV-2-positive and negative patients. No statistically significant difference was detected in the antibiotic susceptibility rates of the microorganisms grown in COVID-19-positive and negative patients in the current study (*p* > 0.05).

Since fastidious bacteria cannot grow on standard media, the isolation of such microorganisms from blood cultures is closely associated with the media used for subculture and incubation conditions [11]. The fastidious bacteria that were isolated in this study show the importance of using additional enriched media and various incubation conditions.

Species-level identification of not only bacteria but also fungi isolated from blood cultures is important in predicting the antifungal resistance of the isolates. As found in the present study, it is noteworthy that *C. auris*, a species that is resistant to numerous antifungal drugs and that can cause fatal outbreaks in the ICU, was reported during the COVID-19 pandemic [18,32,33,34,35]. Although the number of these isolates was low, detection is important in terms of applying the right treatment at the right time, especially considering the special health conditions of the patients in ICUs. In a previous study that investigated fungal colonization in the different body parts of COVID-19 patients hospitalized in the ICU, it was reported that the presence of colonization with non-*albicans Candida* species, which can be associated with treatment failures due to antifungal resistance, was significantly higher and more common in ICU patients compared to non-COVID-19 patients [36].

### Limitations

The present study also has important limitations. Disease duration, the length of hospital stays, treatment of patients, and the rate of readmittances were not included. Whether blood samples are taken in or 48 h after admittance is not known, due to the high workloads during the COVID-19 pandemic. As the present study focused on BSI in COVID-19, data regarding other culture results were not analyzed, which precluded the analysis of other secondary infections such as pneumonia.

## 5. Conclusions

The results of this study show that the most common isolate was MR-CoNS in SARS-CoV-2-positive patients (*p* = 0.028); the detection of *A. baumannii* was more frequent (*p* = 0.004) and the isolation of carbapenem-resistant *K. pneumoniae* was at a higher rate (60% vs. 43%) than in SARS-CoV-2-negative patients (*p* > 0.05), which indicates that paying attention to isolation procedures and the major impact of measures to reduce mortality via reducing the risk of infection should not be disregarded while focusing on the outbreak. The presence of bacterial/fungal agents in bloodstream infections and their antibiotic resistance should still be followed up during a pandemic.

## Figures and Tables

**Table 1:** Distribution of SARS-CoV-2 (+) and SARS-CoV-2 (−) patients according to clinics.

Clinics	Unit	SARS CoV-2 (+) (n:118)	SARS CoV-2 (−) (n:515)	Not Tested (n:19)
Inpatient	Surgical	24	96	-
	Internal	19	69	3
Outpatient	Surgical	-	26	-
	Internal	38	243	14
Intensive Care Unit	Surgical	5	43	1
	Internal	32	38	1

**Table 2:** Distribution of the bacteria and fungi isolated from the blood cultures of SARS-CoV-2-positive and negative patients.

Microorganisms	Total n (%)	SARS- CoV-2 Positive n (%)	SARS- CoV-2 Negative n (%)	SARS- CoV-2 Non-Tested n (%)	Positive vs. Negative	
All microorganisms ^a^	671 (100)	121 (18.0)	531 (79.1)	19 (2.8)	*p*-value	Differences of two proportions 95% CI ^j^
Fermentative Gram-negative rods	252 (37.5)	34 (28,0)	214 (40.3)	4 (21)	0.013 ^h^	(3.2–21.2)
*Escherichia coli*	131 (19.5)	15 (12.4)	113 (21.3)	3 (15.8)	0.026 ^h^	(2.1–5.7)
*Klebsiella pneumoniae*^b^/ CRKP ^c^	80/38 (11.9/5.6)	15/9 (12.4/7.4)	64/28 (12.05/5.3)	1/1 (5.3/5.3)	0.917 ^b,h^ 0.353 ^c,h^	(−6.1–0.8)
						(−2.9–0.5)
*Klebsiella oxytoca*	5 (0.7)	0 (0.0)	5 (0.9)	-	0.590 ^i^	(0.1–1.7)
*Enterobacter* spp.	7 (1.1)	1 (0.8)	6 (1.1)	-	1.000 ^i^	(−1.5–2.1)
*Proteus mirabilis*	13 (1.9)	1 (0.8)	12 (2.3)	-	0.480 ^i^	(−0.5–3.5)
Non-Fermentative Gram-negative rods	66 (9.8)	16 (13.2)	48 (9)	2 (10.5)	0.163 ^h^	(−1.7–10.1)
*Pseudomonas aeruginosa*	25 (3.7)	2 (1.7)	23 (4.3)	-	0.199 ^i^	(−0.2–5.5)
*Pseudomonas* spp.	5 (0.7)	2 (1.7)	3 (0.6)	-	0.233 ^i^	(−0.7–2.8)
*Acinetobacter baumannii*	14 (2.1)	7 (5.8)	6 (1.1)	1 (5.3)	0.004 ^i^	(2.0–7.4)
*Stenotrophomonas maltophilia*	7 (1.05)	1 (0.8)	6 (1.1)	-	1.000 ^i^	(−1.5–2.1)
Gram-positive cocci	300 (44.7)	59 (48.8)	231 (40.1)	10 (52.6)	0.294 ^h^	(−4.5–15.1)
MRSA ^d^	27 (4.0)	3 (2.5)	24 (4.5)	-	0.309 ^h^	(−1.3–5.3)
MSSA ^e^	53 (7.9)	7 (5.8)	43 (8.1)	3 (15.8)	0.388 ^h^	(−2.5–7.1)
MR-CoNS ^f^	102 (15.2)	26 (21.5)	72 (13.5)	4 (21.1)	0.028 ^h^	(1.0–15.0)
MS-CoNS ^g^	49 (7.3)	12 (9.9)	35 (6.6)	2 (10.5)	0.202 ^h^	(−1.8–8.4)
*Enterococcus faecalis*	12 (1.8)	2 (1.7)	10 (1.9)	-	1.000 ^i^	(−2.3–2.8)
*Enterococcus faecium*	12 (1.8)	3 (2.5)	9 (1.7)	-	0.474 ^i^	(−1.9–3.5)
*Enterococcus* spp.	16 (2.4)	2 (1.7)	14 (2.6)	-	0.749 ^i^	(−1.7–3.6)
Alpha hemolytic streptococci	7 (1.05)	0 (0.0)	7 (1.3)	-	0.359 ^i^	(0.3–2.3)
Fungi	38 (5.7)	8 (6.6)	27 (5.1)	3 (15.8)	0.501 ^h^	(−3.0–6.0)
*Candida albicans*	15 (2.2)	2 (1.7)	11 (2.1)	2 (10.5)	1.000 ^i^	(−2.1–3.0)
*Candida parapsilosis* complex	7 (1.05)	1 (0.8)	6 (1.1)	-	1.000 ^i^	(−1.5–2.1)
*Candida tropicalis*	6 (0.9)	0 (0.0)	5 (0.9)	1 (5.3)	0.590 ^i^	(0.1–1.7)

^a^. Numbers of ≤ 4 isolates were not specified in the table: Fermentative Gram-negative rods: *Serratia marcescens* (n:1), *Serratia* spp. (n:2), *Citrobacter* spp. (n:3), *Citrobacter koseri* (n:1), *Morganella morganii* (n:3), *Raoultella planticola* (n:1), *Aeromonas* spp. (n:1), and *Salmonella* Enteritidis (n:4). Non-fermentative Gram-negative rods: *Pseudomonas stutzeri* (n:1), *Acinetobacter lwoffii* (n:1), *Acinetobacter* spp. (n:4), *Rhizobium radiobacter* (n:2), *Achromobacter xylosoxidans* (n:1), *Ochrobactrum anthropi* (n:1), *Sphingomonas paucimobilis* (n:2), *Burkholderia cepacia* (n:1), and *Pandoraea* spp (n:1). Non-fermentative Gram-negative rod (n:1); Gram-positive cocci: *Enterococcus avium* (n:4), *Enterococcus gallinarum* (n:3), *Streptococcus pneumoniae* (n:4), *Streptococcus agalactiae* (n:3), *Streptococcus gallolyticus* (n:2), *Streptococcus equi* (n:1), and *Leuconostoc pseudomesenteroides* (n:2). Beta hemolytic streptococci (n:2). Non-hemolytic streptococci (n:1). Gram-positive rods: *Corynebacterium jeikeium* (n:1), *Corynebacterium striatum* (n:1), and *Lactobacillus casei* (n:1). Other bacteria: *Listeria monocytogenes* (n:2), *Campylobacter coli* (n:1), *Campylobacter jejuni* (n:1), and *Moraxella nonliquefaciens* (n:1). Anaerobic bacteria: *Bacteroides fragilis* (n:1), *Bacteroides* spp. (n:1), *Prevotella* spp. (n:2), *Clostridium clostridioforme* (n:1), and *Fusobacterium nucleatum* (n:1), anaerobic Gram-positive rod (n:1). Fungi: *Candida kefyr* (n:2), *Candida glabrata* complex (n:2), *Candida metapsilosis* (n:1), *Candida krusei* (n:1), *Candida auris* (n:1), *Kodamaea ohmeri* (n:1), *Cryptococcus neoformans* (n:1), and *Rhodotorula* spp. (n:1); ^b^. All *K. pneumoniae* isolates; ^c^. CRKP: Carbapenem-resistant *Klebsiella pneumoniae*; ^d^. MRSA: Methicillin-resistant *Staphylococcus aureus*; ^e^. MSSA: Methicillin-sensitive *Staphylococcus aureus*; ^f^. MR-CoNS: Methicillin-resistant coagulase-negative staphylococci; ^g^. MS-CoNS: Methicillin-sensitive coagulase-negative staphylococci; ^h^. Pearson’s chi-square test; ^i^. Fisher’s exact test; ^j^. CI: confidence interval.

**Table 3:** Antifungal resistance profiles of fungi isolated from the blood cultures of SARS-CoV-2-positive and negative patients.

Fungi	Patients		Antifungal MIC, µg/mL						
Species (Tested *n*/Total *n*)	Strain No	SARS-CoV-2 Status	Fluconazole	Posaconazole	Voriconazole	Itraconazole	Amphotericin B	Caspofungin	Anidulafungin
*Candida albicans* (7/15)	1	Positive	2 (S)	0.064 ᵃ (NWT)	0.25 (I)	-	0.5 ᵃ (WT)	0.016 (S)	0.012 (S)
	2	Negative	2 (S)	0.064 ᵃ (NWT)	0.047(S)	-	-	-	-
	3	Not tested	0.75 (S)	-	-	-	-	0.5 (I)	-
	4	Negative	2 (S)	-	-	-	0.25 ᵃ (WT)	0.5 (I)	0.012 (S)
	5	Positive	1.5 (S)	-	-	-	-	-	-
	6	Negative	-	0.25 ᵃ (NWT)	1 (R)	-	-	0.065 (S)	-
	7	Negative	0.125 (S)	-	-	-	0.047 ᵃ (WT)	0.096 (S)	0.003 (S)
*Candida parapsilosis* complex (3/7)	1	Negative	0.75 (S)	-	-	-	0.25 ᵃ (WT)	0.75 (S)	-
	2	Negative	>256 (R)	0.19 ᵃ (WT)	0.5 (I)	2 ^c^	0.75 ᵃ (WT)	0.38 (S)	0.75 (S)
	3	Negative	24 (R)				0.25 ᵃ (WT)	0.75 (S)	
*Candida tropicalis* (2/6)	1	Negative	0.5 (S)	-	0.008 (S)	-	0.25 ᵃ (WT)	0.094 (S)	0.008 (S)
	2	Negative	0.5 (S)	-	-	-	0.25 ᵃ (WT)	-	0.008 (S)
*Candida glabrata* complex (1/2)		Positive	1.5 (SDD)	-	0.032 ᵃ (WT)	-	-	0.25 (I)	-
*Candida auris* (1/1) ^b^		Positive	>256 (R)	0.016^c^	0.19 ^c^	0.19 ^c^	3 (R)	1 (S)	0.094 (S)
*Cryptococcus neoformans* (1/1)		Negative	8 ᵃ (WT)	-	-	-	0.5 ᵃ (WT)	-	-

MIC: Minimum inhibitory concentration; n: number of isolates; ^a^: MICs were evaluated according to epidemiological cut-off values (ECVs); ^b^: MICs were evaluated according to the breakpoints determined by the CDC; ^c^: there are no clinical breakpoints or ECVs; S: susceptible; I: intermediate; R: resistant; SDD: susceptible-dose dependent; WT: wild type; NWT: non-wild type.

**Table 4:** Demographic information of the patients from whom some microorganisms were isolated.

	Microorganisms (n)						
Demographic Information	MR-CoNS ^a^ (n:102)	MS-CoNS ^b^ (n:49)	*E. coli* (n:131)	*K. pneumoniae* (n:80)	*A. baumannii* (n:14)	*C. albicans* (n:15)	*C. glabrata complex* (n:2)
SARS-CoV-2 (+)	26	12	15	64	7	3	2
Inpatient	9	4	3	21	1	2	2
Outpatient	6	5	9	29	0	0	0
ICU	11	3	3	14	6	1	0
SARS-CoV-2 (−)	72	35	113	15	6	10	0
Inpatient	34	18	23	9	3	8	0
Outpatient	25	13	81	2	0	1	0
ICU	13	4	10	4	3	1	0
SARS-CoV-2—not tested	4	2	3	1	1	2	0
Inpatient	1	0	0	0	0	1	0
Outpatient	3	2	3	0	0	1	0
ICU	0	0	0	1	1	0	0
Median age	63.85	63.42	62.69	60.78	63.71	68.4	71
Gender identity							
Male	44	25	74	44	10	8	1
Female	58	24	57	36	4	7	1
Oncology patient	39	11	40	26	3	6	0
Hematologic malignancy	7	6	13	3	2	2	0
Hypertension	33	17	36	33	6	6	1
Diabetes mellitus	22	11	27	19	3	3	1
Coronary artery disease	0	0	10	0	0	2	0
COPD ^c^	9	2	0	0	0	3	0
COVID-19 pneumonia	22	14	24	12	5	2	0

n: Number of patients, ^a^. MR-CoNS: methicillin-resistant coagulase-negative staphylococci; ^b^. MS-CoNS: methicillin-sensitive coagulase-negative staphylococci; ^c^. COPD: chronic obstructive pulmonary disease.

**Table 5:** Distribution of the polymicrobial growths detected in patients ^a^.

	Clinic/Units		Microorganism
SARS-CoV-2 (+) [n:8]	Inpatient (n:3)	Surgical (n:1)	*Candida krusei, Kodamea ohmeri*
		Internal (n:2)	*Proteus mirabilis, Escherichia coli*
			*Klebsiella pneumoniae, Candida glabrata* complex
	Outpatient (n:4)	Surgical (n:1)	*Candida glabrata* complex, *Candida albicans*
		Internal (n:3)	*Streptococcus pneumoniae, Escherichia coli*
			*Candida kefyr, Enterococcus gallinarum, Enterococcus faecium*
			*Escherichia coli, Enterobacter* spp.
	Intensive Care Unit (n:1)	Surgical (n:0)	*-*
		Internal (n:1)	*Enterococcus* spp., *Candida albicans*
SARS-CoV-2 (-) [n:9]	Inpatient (n:3)	Surgical (n:2)	*Candida albicans, Candida parapsilosis*
			*Escherichia coli, Candida parapsilosis*
		Internal (n:1)	*Pseudomonas aeruginosa, Acinetobacter* spp.
	Outpatient (n:5)	Surgical (n:1)	*Citrobacter* spp., *Klebsiella oxytoca*
		Internal (n:4)	*Raoultella planticola, Escherichia coli*
			*Klebsiella pneumoniae, Enterococcus* spp.
			*Enterococcus* spp., *Escherichia coli*, MSSA ^b^
			*Enterococcus* spp., *Escherichia coli*
	Intensive Care Unit (n:1)	Surgical (n:0)	*-*
		Internal (n:1)	*Proteus mirabilis, Klebsiella pneumoniae*

^a^: Difference in proportion between SARS-CoV-2-positive and negative patients for polymicrobial growth (*p* = 0.006), ^b^: methicillin-sensitive *Staphylococcus aureus.*

## Data Availability

All the data are included in the article by excluding personal data that may not comply with GDPR regulations.
